# Spread of a model invasive alien species, the harlequin ladybird *Harmonia axyridis* in Britain and Ireland

**DOI:** 10.1038/sdata.2018.239

**Published:** 2018-10-23

**Authors:** P. M. J. Brown, D. B. Roy, C. Harrower, H. J. Dean, S. L. Rorke, H. E. Roy

**Affiliations:** 1 Applied Ecology Research Group, Department of Biology, Anglia Ruskin University, Cambridge, CB1 1PT, UK; 2 Biological Records Centre, NERC Centre for Ecology and Hydrology, Wallingford, Oxfordshire, OX10 8BB, UK

**Keywords:** Invasive species, Invasive species, Entomology

## Abstract

Invasive alien species are widely recognized as one of the main threats to global biodiversity. Rapid flow of information on the occurrence of invasive alien species is critical to underpin effective action. Citizen science, i.e. the involvement of volunteers in science, provides an opportunity to improve the information available on invasive alien species. Here we describe the dataset created via a citizen science approach to track the spread of a well-studied invasive alien species, the harlequin ladybird *Harmonia axyridis* (Coleoptera: Coccinellidae) in Britain and Ireland. This dataset comprises 48 510 verified and validated spatio-temporal records of the occurrence of *H. axyridis* in Britain and Ireland, from first arrival in 2003, to the end of 2016. A clear and rapid spread of the species within Britain and Ireland is evident. A major reuse value of the dataset is in modelling the spread of an invasive species and applying this to other potential invasive alien species in order to predict and prevent their further spread.

## Background & Summary

The invasion process for an alien species involves various stages, notably introduction, establishment, increase in abundance and geographic spread^1^. An alien species that spreads and has negative effects (which may be ecological, economic or social) is termed invasive^2,3^. Invasive alien species are widely recognized as one of the main threats to global biodiversity^4–6^. There are a number of international agreements which recognize the threat posed by invasive alien species, which are designated as a priority within the Convention on Biological Diversity Aichi biodiversity target 9 (https://www.cbd.int/sp/targets/rationale/target-9/) and are relevant to many of the Sustainable Development Goals (http://www.un.org/sustainabledevelopment/sustainable-development-goals/). An EU Regulation on invasive alien species came into force on 1 January 2015 (http://ec.europa.eu/environment/nature/invasivealien/index_en.htm) and subsequently a list of invasive alien species of EU concern was adopted for which member states are required to take action to eradicate, manage or prevent entry.

Rapid flow of information on the occurrence of invasive alien species is critical to underpin effective action. There have been few attempts to monitor the spread of invasive alien species systematically from the onset of the invasion process. Citizen science, i.e. the involvement of volunteers in science, provides an opportunity to improve the information available on invasive alien species^7^. Here we describe the dataset created via a citizen science approach to track the spread of a well-studied invasive alien species, the harlequin ladybird *Harmonia axyridis* (Coleoptera: Coccinellidae) in Britain and Ireland. This species was detected very early in the invasion process and a citizen science project was initiated and widely promoted to maximize the opportunity to gather data from the public across Britain and Ireland.

*Harmonia axyridis* was introduced between approximately 1982 and 2003 to at least 13 European countries^8^ as a biological control agent. It was mainly introduced to control aphids that are pests to a range of field and glasshouse crops. From the early 2000s it subsequently spread to many other European countries, including Britain and Ireland. It is native to Asia (including China, Japan, Mongolia and Russia)^9^ and was also introduced in North and South America and Africa^10^. *Harmonia axyridis* was introduced unintentionally to Britain from mainland Europe by a number of pathways: some were transported with produce such as cut flowers, fruit and vegetables; others arrived through natural dispersal (flight) from other invaded regions^11^. To a lesser extent *H. axyridis* also arrived from North America^12^. The major pathways of spread to Ireland were probably natural dispersal (from Britain) and arrival with produce. *Harmonia axyridis* is a eurytopic (generalist) species and may be found on deciduous or coniferous trees, arable and horticultural crops and herbaceous vegetation in a wide range of habitats. It is particularly prevalent in urban and suburban localities (e.g. parks, gardens, and in or on buildings)^13^.

Citizen science approaches to collecting species data are becoming increasingly popular and respected^14^. Advances in communication and digital technologies (e.g. online recording via websites and smartphone applications; digital photography) have increasingly enabled scientists to collect and verify large datasets of species information^15^. For a few species groups, including ladybirds, verification to species is possible if a reasonably good photograph of the animal is available. In late 2004, shortly after the first *H. axyridis* ladybird record was reported, funding was acquired from Defra and the National Biodiversity Network (NBN) to set up and trial an online recording scheme for ladybirds, and *H. axyridis* in particular. Thus, the online Harlequin Ladybird Survey and UK Ladybird Survey were launched in March 2005.

The surveys have been very successful in gaining records from the public since 2005. Innovations such as the launch of a free smartphone application (iRecord Ladybirds) in 2013 helped to maintain the supply of records. The dataset here comprises species records of *H. axyridis* in various life stages (larva, pupa or adult) from Britain and Ireland over the period 2003 to 2016.

A major reuse value of the dataset is in modelling the spread of an invasive species and applying this to other potential invasive alien species in order to predict and prevent their further spread. The time period of the study captures the initial fast spread of *H. axyridis* (principally from 2004 to 2009) plus a further substantial period (2010 to 2016) in which the distribution of the species altered relatively little, despite many further records being received.

## Methods

This dataset (Data Citation 1) comprises 48 510 spatio-temporal records of the occurrence of *H. axyridis* in Britain and Ireland, from first arrival in 2003, to the end of 2016. For its type it is thus an unusually substantial dataset. Whilst the records were collated and verified by the survey organizers, the records themselves were provided by members of the public in Britain and Ireland. Uptake to the Harlequin Ladybird Survey was undoubtedly assisted by the pre-existence of the Coccinellidae Recording Scheme (now the UK Ladybird Survey), supported by the Biological Records Centre (within NERC Centre for Ecology & Hydrology)^16^.

Reflecting the general diversification of citizen science through innovative use of technology^17^, high levels of public access to the internet and digital photography enabled an online survey form to be established for *H. axyridis* in Britain and Ireland. The Harlequin Ladybird Survey (www.harlequin-survey.org) was one of the first online wildlife surveys in Britain and Ireland. It was launched in March 2005 in response to the first report of *H. axyridis* in Britain, in September 2004^18^. The Harlequin Ladybird Survey benefited from high levels of media interest, and members of the public showed great willingness to look for *H. axyridis*, and to register their sightings with the survey^13^. There are only three records from 2003 and no earlier records have been received, supporting the case that the earliest records in the dataset represent the onset of the invasion process for this species. Indeed *H. axyridis* has a relatively high detectability (e.g.^19^) and rapid reproductive rate, so is unlikely to have arrived unnoticed.

Each record represents a verified sighting of *H. axyridis* on a given date (or range of dates) and comprises one or more individual ladybirds observed from one or more life stages (larva, pupa, adult). Records are from Britain (England, Wales and Scotland, including offshore islands), Ireland (both Northern Ireland and the Republic of Ireland), the Isle of Man and the Channel Islands (primarily Guernsey and Jersey) and are mainly from the period 2004 to 2016. The earliest record of *H. axyridis* in Britain was initially thought to be from 3 July 2004, but three earlier records (from 2003) were received retrospectively. The data records represent species presence and there are no absence data available.

The majority of the records were received from members of the public via online recording forms (at www.harlequin-survey.org or www.ladybird-survey.org) (Supplementary Figure 1) or via smartphone apps (iRecord Ladybirds or iRecord - www.brc.ac.uk/irecord) (Supplementary Figure 2), with some records (especially in earlier years) received by post. Other records, particularly from amateur expert^16^ coleopterists and other naturalists, were received in spreadsheets.

The spatial resolution of the records is variable. Many include an Ordinance Survey grid reference (converted to latitude and longitude), enabling resolution to 100 metres or less, but many others were derived at 1 km resolution from a UK postal code (UK Government Schemas and Standards, http://webarchive.nationalarchives.gov.uk/20101126012154/http://www.cabinetoffice.gov.uk/govtalk/schemasstandards/e-gif/datastandards/address/postcode.aspx). The option on the online recording form to enter the location via a UK postal code was provided to make the entry of records easier for members of the public unfamiliar with grid referencing systems. Whilst the resolution is thus reduced for these records, the reduction in user error (e.g. the problem of grid reference eastings and northings being transposed) is an advantage^20^. The postal code method was applicable for sightings of *H. axyridis* made within 200 metres of a specified postal code, so could not be used for a minority of records where the ladybird was seen in a remote semi-natural habitat. The spatial resolution of the records tended to increase over time, as the number of records received via the smartphone apps increased, and these records generally have GPS-generated latitudes and longitudes.

## Data Records

### Repository

The dataset is freely available for download from the Environmental Information Data Centre (EIDC) catalogue (Data Citation 1). The dataset is provided as a single tab-delimited text file, with each line representing a single record.

### Constituents of Species Records

Each species record includes 19 fields (Table 1).

### Figures and Tables

**Figure 1:**
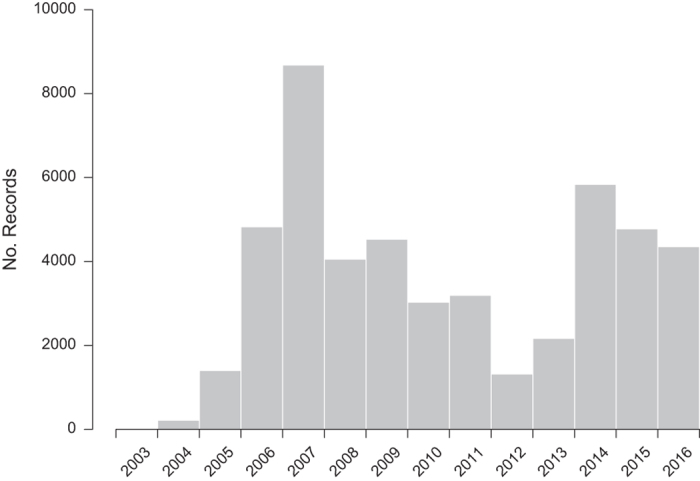
The number of verified *Harmonia axyridis* records received for Britain, the Channel Islands and Ireland by year, from 2003 to 2016.

**Figure 2:**
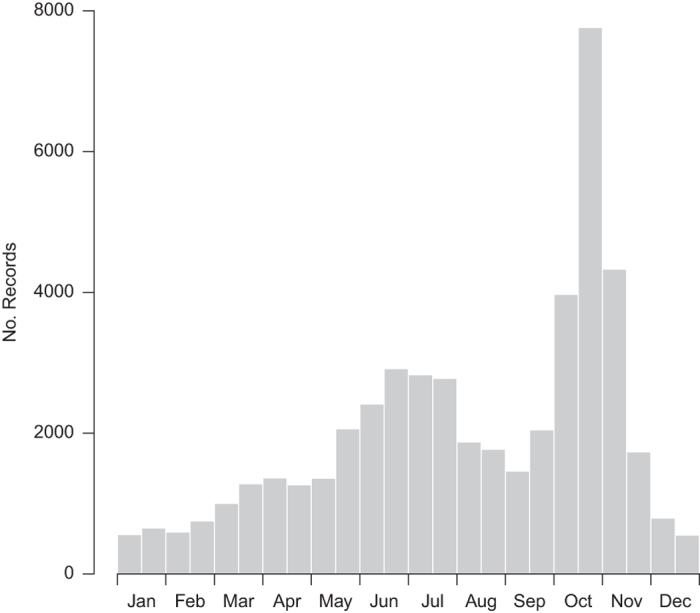
The number of verified *Harmonia axyridis* records received for Britain, the Channel Islands and Ireland by month, from 2003 to 2016.

**Figure 3:**
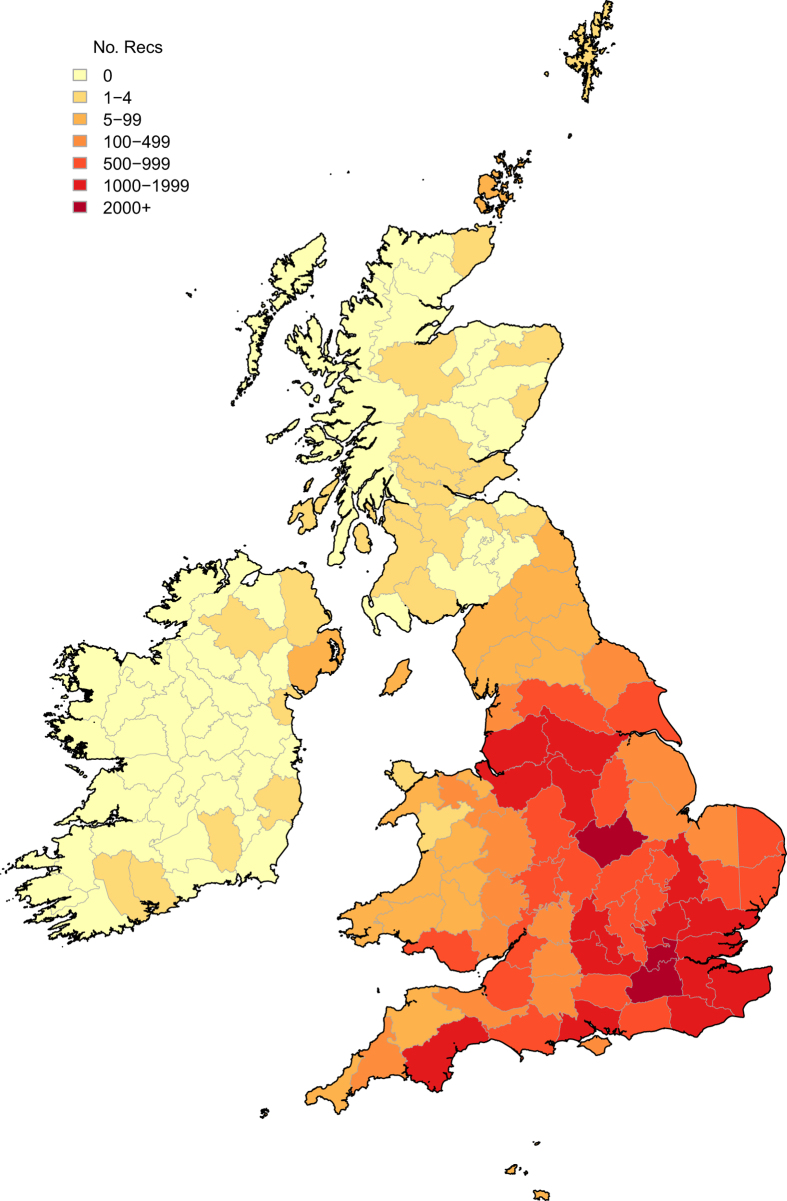
The number of verified *Harmonia axyridis* records received for Britain, the Channel Islands and Ireland, split by Vice County, from 2003 to 2016.

**Figure 4:**
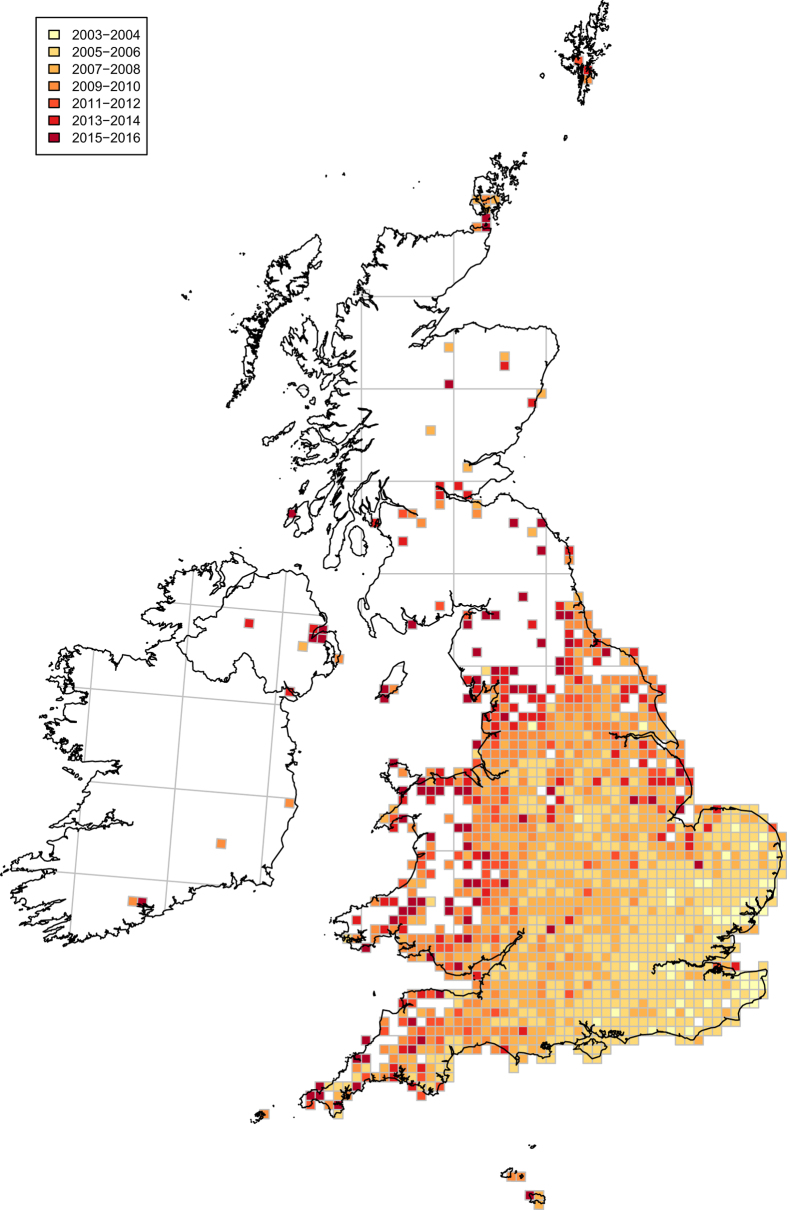
The spread and distribution in 10 km squares in Britain, the Channel Islands and Ireland of *Harmonia axyridis* from 2003 to 2016. NB where *H. axyridis* was recorded in a square in multiple time periods, the older time period overlays the newer one(s).

The figures and tables here show a summary of the dataset, notably the number of verified *H. axyridis* records received by year (Fig. 1), by month (Fig. 2), by vice county (Fig. 3 and Table 2 (available online only)) and the spread of *H. axyridis* in Britain, the Channel Islands and Ireland from 2003 to 2016 (Fig. 4).

## Technical Validation

### Record Verification

Verification of the records was made by the survey organizers (led by HER and PMJB but also including others) on receipt of either a photograph or ladybird specimen. The records received from amateur expert coleopterists and other naturalists are regarded as accurate (i.e. without the survey organizers seeing a photograph or specimen) and have been included in the dataset. Many further online records were received that remain unverified (i.e. no photograph or specimen was sent, or the photograph was of insufficient quality to enable identification) or were verified as another species. All such unverified or inaccurate records are excluded from this dataset. For discussion of these issues (partly relating to our dataset) see^21^. Verified records were regularly uploaded to the NBN Gateway (now the NBN Atlas - https://nbnatlas.org/). There the records could be viewed via online maps, which helped to encourage further recording.

### Recording Intensity

Recording intensity by the public was not consistent over time and was influenced by media coverage, publicity events by the survey organizers, and other factors. The number of records in a period is also influenced by weather conditions and seasonality: the main peak in record numbers each year tended to be from late October to early November, the period in which *H. axyridis* generally moves to indoor overwintering sites (hence this is when many people first notice the species in their homes).

There is also spatial variability in recording intensity: more records come from areas with high densities of people (Fig. 3). Across Britain and Ireland there were a number of particularly active local groups or individuals which contributed hotspots of recorder activity, e.g. London. To many recorders, juvenile stages (especially pupae and early instar larvae) were less noticeable and more difficult to identify than the adult stage, thus limiting their recording. The possibility of a reporting bias towards sightings early in the season also exists (i.e. some recorders may have reported their first sighting of *H. axyridis*, but not subsequent sightings). In order to minimize this effect, the importance of recording multiple sightings was stressed to recorders. The peaks in record numbers observed late in each year also suggest that any effect of this potential bias was minor. There is probably a further minor temporal bias towards recording on some days of the week (e.g. weekend days) more than others.

### Technical Validation

In addition to the expert verification detailed above, each record has also undergone a series of validation checks that are designed to highlight other potential issues with the data. Checks were performed on the date information supplied with the record to ensure that both the start and/or end dates supplied are in recognized formats, are valid dates, are in the past or present (e.g. no future dates), and where both supplied that the start date is prior or equal to the end date. The location information is also checked to ensure that the supplied grid reference is in a recognized format and is a valid grid reference and that the supplied grid reference is from a 10 km and/or 1 km square that contains land. If other location fields were supplied with the grid reference (such as 10 km grid reference, vice county, tetrad or quadrant codes, etc) they were cross-checked to ensure consistency.

## Additional information

**How to cite this article**: Brown, P. M. J. *et al*. Spread of a model invasive alien species, the harlequin ladybird *Harmonia axyridis* in Britain and Ireland. *Sci. Data*. 5:180239 doi: 10.1038/sdata.2018.239 (2018).

**Publisher’s note**: Springer Nature remains neutral with regard to jurisdictional claims in published maps and institutional affiliations.

## Supplementary Material



Supplementary Figures

## Figures and Tables

**Table 1:** The fields contained in each *Harmonia axyridis* species record in the database, with a descriptor for each.

**Field**	**Descriptor**
STARTDATE	Start data for the record (DD/MM/YYYY).
ENDDATE	End date for the record (DD/MM/YYYY).
GRIDREF	The grid reference specifying the location of the observation at the fullest precision available. The grid reference is either Ordnance Survey British (OSGB), Ordnance Survey of Ireland (OSI), or a truncated version of the Military Grid Reference System (MGRS) depending on the location of the record. Records from Britain use OSGB, from Ireland use OSI, and from the Channel Islands use MGRS. OSI grid references are distinguished by having a single letter at the start (e.g. N48), while OSGB and truncated MGRS grid references both start with 2 letters (e.g. SP30), those that start WA or WV belonging to the truncated MGRS and the rest to OSGB. The truncated MGRS grid references used here for the Channel Islands simply omit the zone number and 100 km square code from the start of the grid references as all squares within the Channel Islands are within the same zone and 100 km square 30U (e.g. WV6548 in this dataset refers to MGRS 1 km grid reference 30UWV6548).
PRECISION	The precision of the grid reference (in metres) given in GRIDREF field.
VC	The Watsonian vice county code (https://en.wikipedia.org/wiki/Vice-county). Codes 1 to 112 represent the vice counties of Britain, 113 represents the Channel Islands, and codes 201 to 240 represent Irish vice counties (where 201 = vice county H1, 202 = H2, etc).
LATITUDE	Latitude (WGS 84) for the centre of the grid reference supplied in the GRIDREF field.
LONGITUDE	Longitude (WGS 84) for the centre of the grid reference supplied in the GRIDREF field.
GR_1KM	The grid reference of the 1 km grid square in which the occurrence record lies (only populated if the original grid reference is at 1000 m precision or finer).
LATITUDE_1KM	Latitude (WGS 84) for the centre of the 1 km grid square in which the record occurs.
LONGITUDE_1KM	Longitude (WGS 84) for the centre of the 1 km grid square in which the record occurs.
GR_10KM	The grid reference of the 10 km grid square in which the occurrence record lies.
LATITUDE_10KM	Latitude (WGS 84) for the centre of the 10 km grid square in which the record occurs.
LONGITUDE_10KM	Longitude (WGS 84) for the centre of the 10 km grid square in which the record occurs.
ABUNDANCE	A text field containing any abundance information that was supplied with the record.
FORM_ABUNDANCE	Abundance information relating to color forms, if available
FORM	Color form of the ladybird, if available: *conspicua, spectabilis, succinea.*
STAGE_ABUNDANCE	Abundance information of different life stages, if available.
STAGE	Ladybird life stage: larva, pupa, adult.
RECORDER	ID(s) for recorder name(s) that submitted the record. In the BRC database recorder information is standardized to Surname followed by initials (e.g. Smith, J.). For this reason, the recorder ID relates to a unique standardized name and not an individual. A single ID can refer to multiple individuals and a single individual can also be associated with multiple recorder IDs.

**Table 2:** The number of *Harmonia axyridis* records in the dataset, listed by vice county and region (England, Wales, Isle of Man, Scotland, Channel Islands or Ireland).

**Vice County Number**	**Vice County Name**	**Region**	**Number of Records in Dataset**
1	West Cornwall with Scilly	England	45
2	East Cornwall	England	101
3	South Devon	England	1015
4	North Devon	England	76
5	South Somerset	England	357
6	North Somerset	England	786
7	North Wiltshire	England	334
8	South Wiltshire	England	409
9	Dorset	England	638
10	Isle of Wight	England	212
11	South Hampshire	England	1242
12	North Hampshire	England	705
13	West Sussex	England	775
14	East Sussex	England	1227
15	East Kent	England	1294
16	West Kent	England	1383
17	Surrey	England	3404
18	South Essex	England	1089
19	North Essex	England	1265
20	Hertfordshire	England	1344
21	Middlesex	England	3300
22	Berkshire	England	1654
23	Oxfordshire	England	1360
24	Buckinghamshire	England	983
25	East Suffolk	England	966
26	West Suffolk	England	584
27	East Norfolk	England	986
28	West Norfolk	England	358
29	Cambridgeshire	England	1388
30	Bedfordshire	England	549
31	Huntingdonshire	England	564
32	Northamptonshire	England	550
33	East Gloucestershire	England	498
34	West Gloucestershire	England	834
35	Monmouthshire	Wales	212
36	Herefordshire	England	123
37	Worcestershire	England	930
38	Warwickshire	England	976
39	Staffordshire	England	627
40	Shropshire	England	470
41	Glamorganshire	Wales	795
42	Breconshire	Wales	35
43	Radnorshire	Wales	17
44	Carmarthenshire	Wales	40
45	Pembrokeshire	Wales	13
46	Cardiganshire	Wales	34
47	Montgomeryshire	Wales	50
48	Merionethshire	Wales	4
49	Caernarvonshire	Wales	33
50	Denbighshire	Wales	118
51	Flintshire	Wales	73
52	Anglesey	Wales	4
53	South Lincolnshire	England	325
54	North Lincolnshire	England	477
55	Leicestershire (with Rutland)	England	2163
56	Nottinghamshire	England	970
57	Derbyshire	England	1048
58	Cheshire	England	1137
59	South Lancashire	England	1547
60	West Lancashire	England	289
61	South-east Yorkshire	England	663
62	North-east Yorkshire	England	330
63	South-west Yorkshire	England	1667
64	Mid-west Yorkshire	England	730
65	North-west Yorkshire	England	36
66	County Durham	England	87
67	South Northumberland	England	19
68	North Northumberland	England	5
69	Westmorland (with Furness)	England	43
70	Cumberland	England	8
71	Isle of Man	Isle of Man	39
72	Dumfriesshire	Scotland	0
73	Kirkcudbrightshire	Scotland	3
74	Wigtownshire	Scotland	0
75	Ayrshire	Scotland	2
76	Renfrewshire	Scotland	1
77	Lanarkshire	Scotland	4
78	Peeblesshire	Scotland	0
79	Selkirkshire	Scotland	0
80	Roxburghshire	Scotland	0
81	Berwickshire	Scotland	4
82	East Lothian	Scotland	0
83	Midlothian	Scotland	4
84	West Lothian	Scotland	0
85	Fifeshire	Scotland	1
86	Stirlingshire	Scotland	2
87	West Perthshire	Scotland	2
88	Mid Perthshire	Scotland	4
89	East Perthshire	Scotland	0
90	Angus	Scotland	0
91	Kincardineshire	Scotland	3
92	South Aberdeenshire	Scotland	0
93	North Aberdeenshire	Scotland	3
94	Banffshire	Scotland	0
95	Moray	Scotland	0
96	East Inverness-shire	Scotland	3
97	West Inverness-shire	Scotland	0
98	Argyllshire	Scotland	0
99	Dunbartonshire	Scotland	0
100	Clyde Isles	Scotland	1
101	Kintyre	Scotland	0
102	South Ebudes	Scotland	1
103	Mid Ebudes	Scotland	0
104	North Ebudes	Scotland	0
105	West Ross & Cromarty	Scotland	0
106	East Ross & Cromarty	Scotland	0
107	East Sutherland	Scotland	0
108	West Sutherland	Scotland	0
109	Caithness	Scotland	2
110	Outer Hebrides	Scotland	0
111	Orkney	Scotland	9
112	Shetland	Scotland	3
113	Channel Islands	Channel Islands	20
201	South Kerry	Ireland	0
202	North Kerry	Ireland	0
203	West Cork	Ireland	0
204	Mid-Cork	Ireland	4
205	East Cork	Ireland	1
206	Waterford	Ireland	0
207	South Tipperary	Ireland	0
208	Limerick	Ireland	0
209	Clare	Ireland	0
210	North Tipperary	Ireland	0
211	Kilkenny	Ireland	1
212	Wexford	Ireland	0
213	Carlow	Ireland	0
214	Laois	Ireland	0
215	South-east Galway	Ireland	0
216	West Galway	Ireland	0
217	North-east Galway	Ireland	0
218	Offaly	Ireland	0
219	Kildare	Ireland	0
220	Wicklow	Ireland	1
221	Dublin	Ireland	0
222	Meath	Ireland	0
223	Westmeath	Ireland	0
224	Longford	Ireland	0
225	Roscommon	Ireland	0
226	East Mayo	Ireland	0
227	West Mayo	Ireland	0
228	Sligo	Ireland	0
229	Leitrim	Ireland	0
230	Cavan	Ireland	0
231	Louth	Ireland	1
232	Monaghan	Ireland	0
233	Fermanagh	Ireland	0
234	East Donegal	Ireland	0
235	West Donegal	Ireland	0
236	Tyrone	Ireland	1
237	Armagh	Ireland	0
238	Down	Ireland	11
239	Antrim	Ireland	3
240	Londonderry	Ireland	0
