# Supplementary material for: Spread of a model invasive alien species, the harlequin ladybird Harmonia axyridis in Britain and Ireland
Source: Sci Data. 2018 Oct 23;5:180239. doi: 10.1038/sdata.2018.239 (PMC6198752; doi:10.1038/sdata.2018.239)
Supplement: Supplementary Figures [file sdata2018239-s2.pdf]

Supplementary Information

Table of Contents:

Supplementary Figure 1. Online recording pages from the Harlequin Ladybird Survey website (www.harlequin-survey.org); Page 2.

Supplementary Figure 2. Screenshots from the iRecord Ladybirds smartphone application (app) used to record ladybird species, with *Harmonia axyridis* used as an example throughout; Page 4.

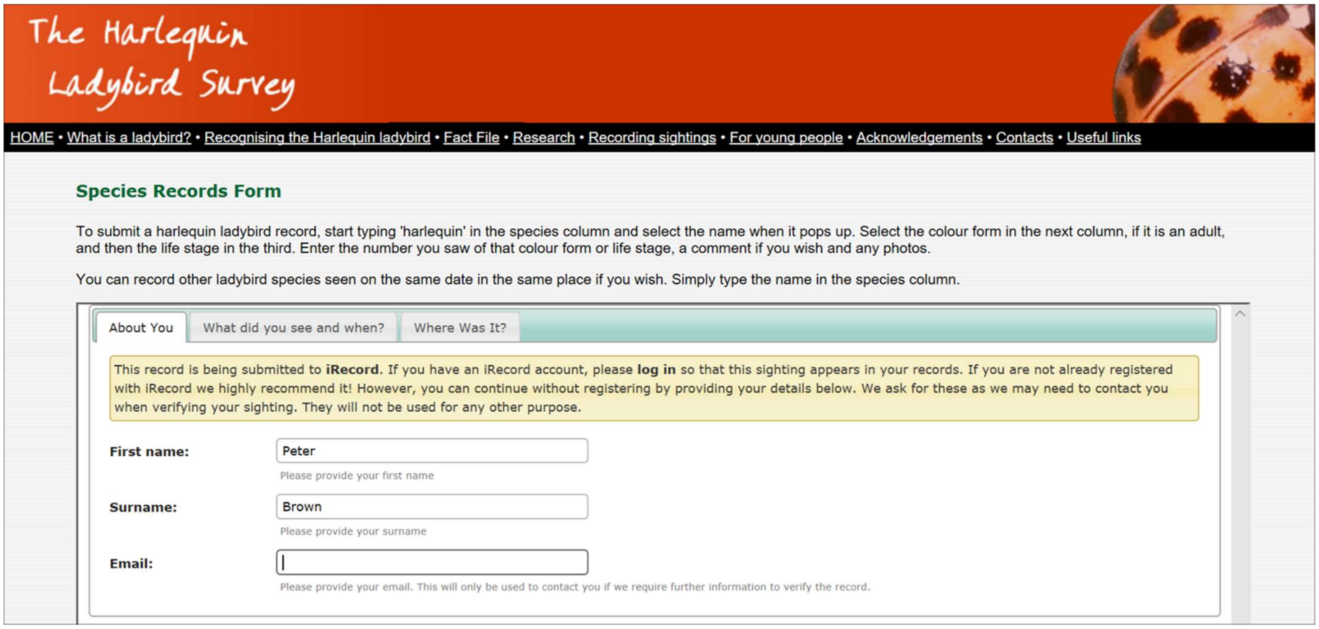

The Harlequin Ladybird Survey

HOME • What is a ladybird? • Recognising the Harlequin ladybird • Fact File • Research • Recording sightings • For young people • Acknowledgements • Contacts • Useful links

Species Records Form

To submit a harlequin ladybird record, start typing 'harlequin' in the species column and select the name when it pops up. Select the colour form in the next column, if it is an adult, and then the life stage in the third. Enter the number you saw of that colour form or life stage, a comment if you wish and any photos.

You can record other ladybird species seen on the same date in the same place if you wish. Simply type the name in the species column.

About You | What did you see and when? | Where Was It?

This record is being submitted to **iRecord**. If you have an iRecord account, please **log in** so that this sighting appears in your records. If you are not already registered with iRecord we highly recommend it! However, you can continue without registering by providing your details below. We ask for these as we may need to contact you when verifying your sighting. They will not be used for any other purpose.

First name: Peter

Please provide your first name

Surname: Brown

Please provide your surname

Email:

Please provide your email. This will only be used to contact you if we require further information to verify the record.

a.

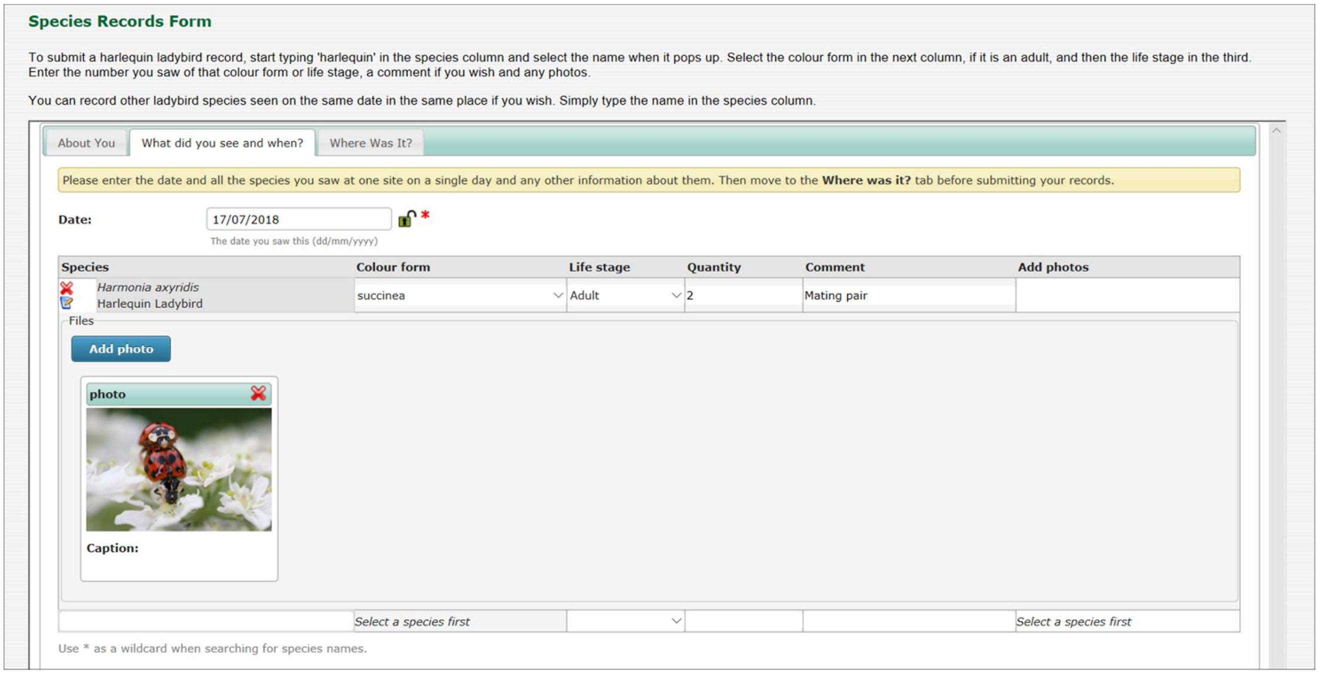

Species Records Form

To submit a harlequin ladybird record, start typing 'harlequin' in the species column and select the name when it pops up. Select the colour form in the next column, if it is an adult, and then the life stage in the third. Enter the number you saw of that colour form or life stage, a comment if you wish and any photos.

You can record other ladybird species seen on the same date in the same place if you wish. Simply type the name in the species column.

About You | What did you see and when? | Where Was It?

Please enter the date and all the species you saw at one site on a single day and any other information about them. Then move to the **Where was it?** tab before submitting your records.

Date: 17/07/2018 *

The date you saw this (dd/mm/yyyy)

Species	Colour form	Life stage	Quantity	Comment	Add photos
Harmonia axyridis Harlequin Ladybird	succinea	Adult	2	Mating pair	

Files

Add photo

photo

Caption:

	Select a species first				*Select a species first*

Use * as a wildcard when searching for species names.

b.

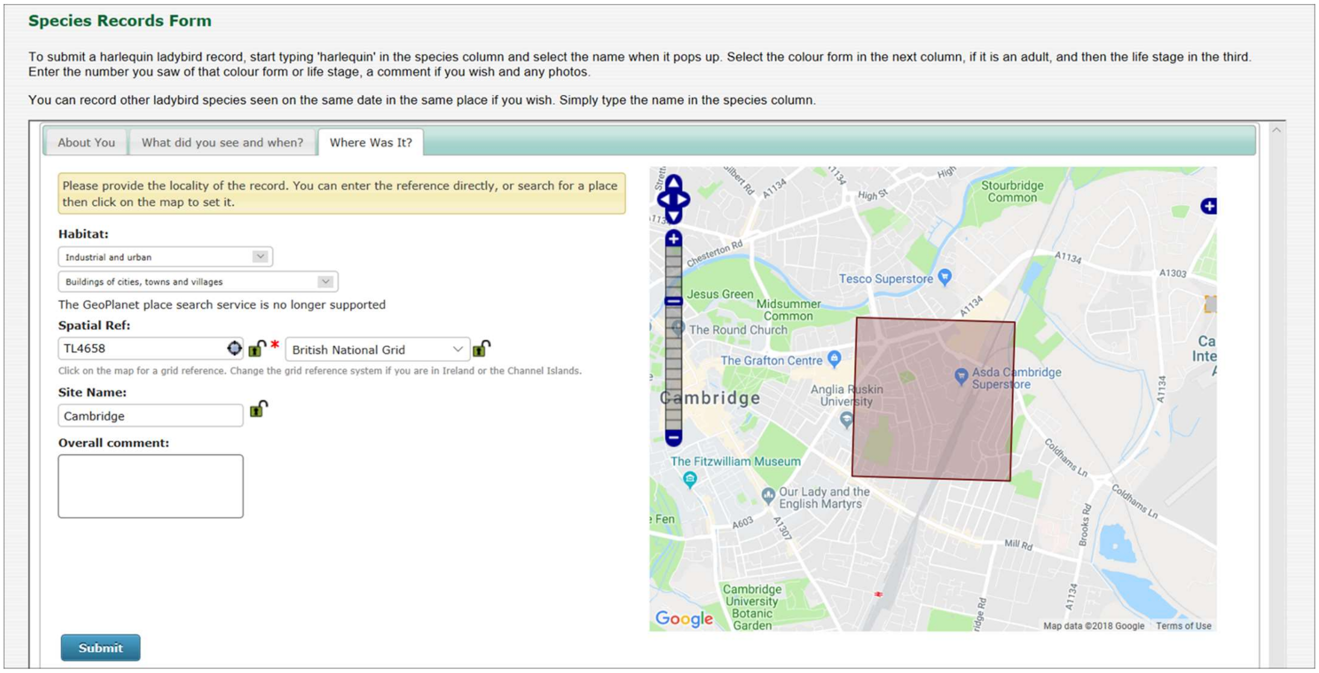

c.

Supplementary Figure 1. Online recording pages from the Harlequin Ladybird Survey website (www.harlequin-survey.org). **a.** First page, requesting basic information about the recorder; **b.** Second page, requesting information on the date of the sighting and on the species seen (including the opportunity to upload one or more photos for verification purposes); **c.** Third page, requesting information on the habitat and location where the species was found, with space for comments (e.g. ecological or behavioural observations made by the recorder). The location may be selected either by entering a grid reference or by clicking on the interactive map.

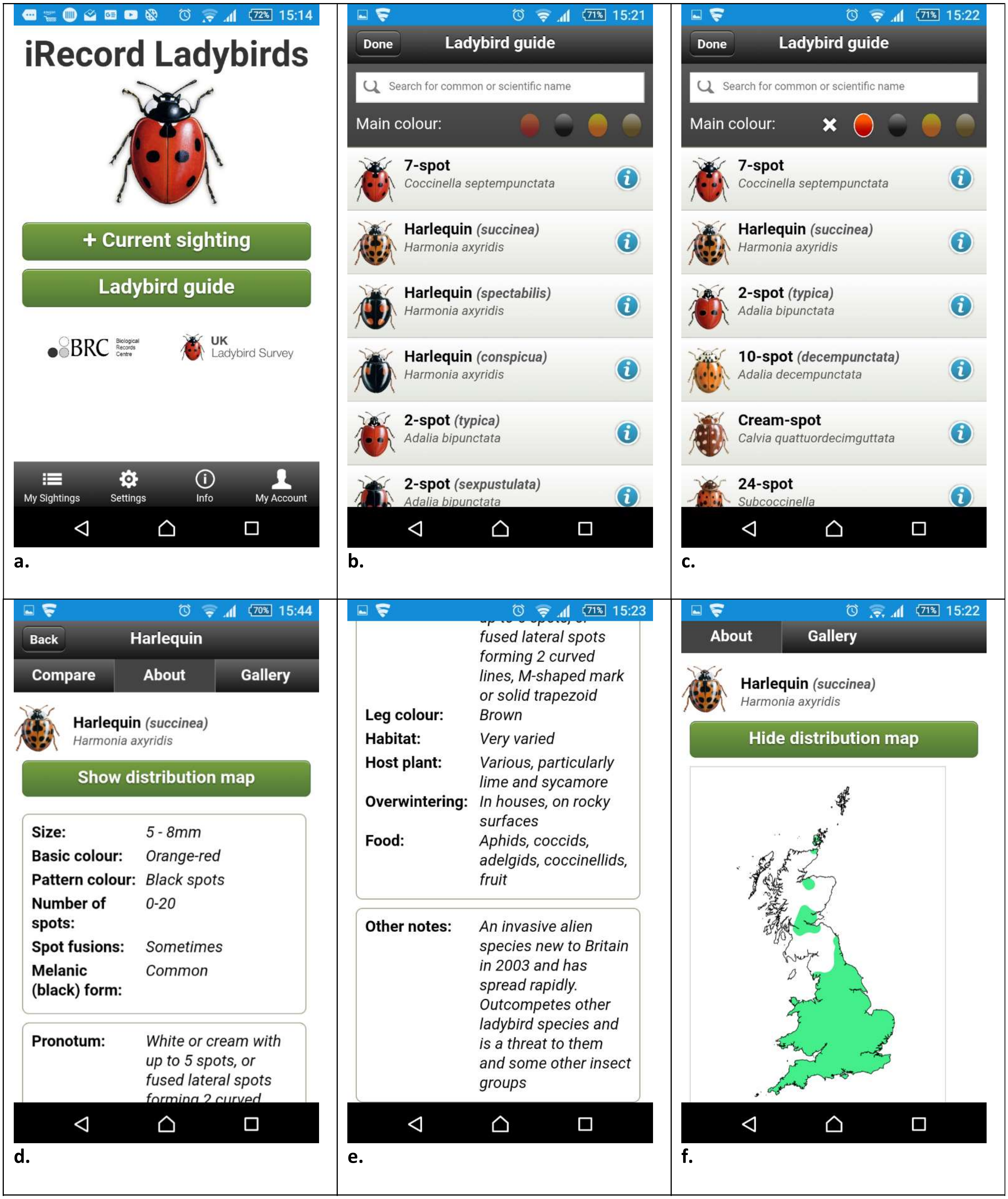
15:14
iRecord Ladybirds
+ Current sighting
Ladybird guide
BRC Biological Records Centre
UK Ladybird Survey
My Sightings
Settings
Info
My Account
a.
15:21
Done
Ladybird guide
Search for common or scientific name
Main colour:
7-spot Coccinella septempunctata
Harlequin (succinea) Harmonia axyridis
Harlequin (spectabilis) Harmonia axyridis
Harlequin (conspicua) Harmonia axyridis
2-spot (typica) Adalia bipunctata
2-spot (sexpustulata) Adalia bipunctata
b.
15:22
Done
Ladybird guide
Search for common or scientific name
Main colour:
7-spot Coccinella septempunctata
Harlequin (succinea) Harmonia axyridis
2-spot (typica) Adalia bipunctata
10-spot (decempunctata) Adalia decempunctata
Cream-spot Calvia quattuordecimguttata
24-spot Subcoccinella
c.
15:44
Back
Harlequin
Compare
About
Gallery
Harlequin (succinea) Harmonia axyridis
Show distribution map
Size: 5 - 8mm
Basic colour: Orange-red
Pattern colour: Black spots
Number of spots: 0-20
Spot fusions: Sometimes
Melanic (black) form: Common
Pronotum: White or cream with up to 5 spots, or fused lateral spots forming 2 curved
d.
15:23
fused lateral spots forming 2 curved lines, M-shaped mark or solid trapezoid
Leg colour: Brown
Habitat: Very varied
Host plant: Various, particularly lime and sycamore
Overwintering: In houses, on rocky surfaces
Food: Aphids, coccids, adelgids, coccinellids, fruit
Other notes: An invasive alien species new to Britain in 2003 and has spread rapidly. Outcompetes other ladybird species and is a threat to them and some other insect groups
e.
15:22
About
Gallery
Harlequin (succinea) Harmonia axyridis
Hide distribution map
f.

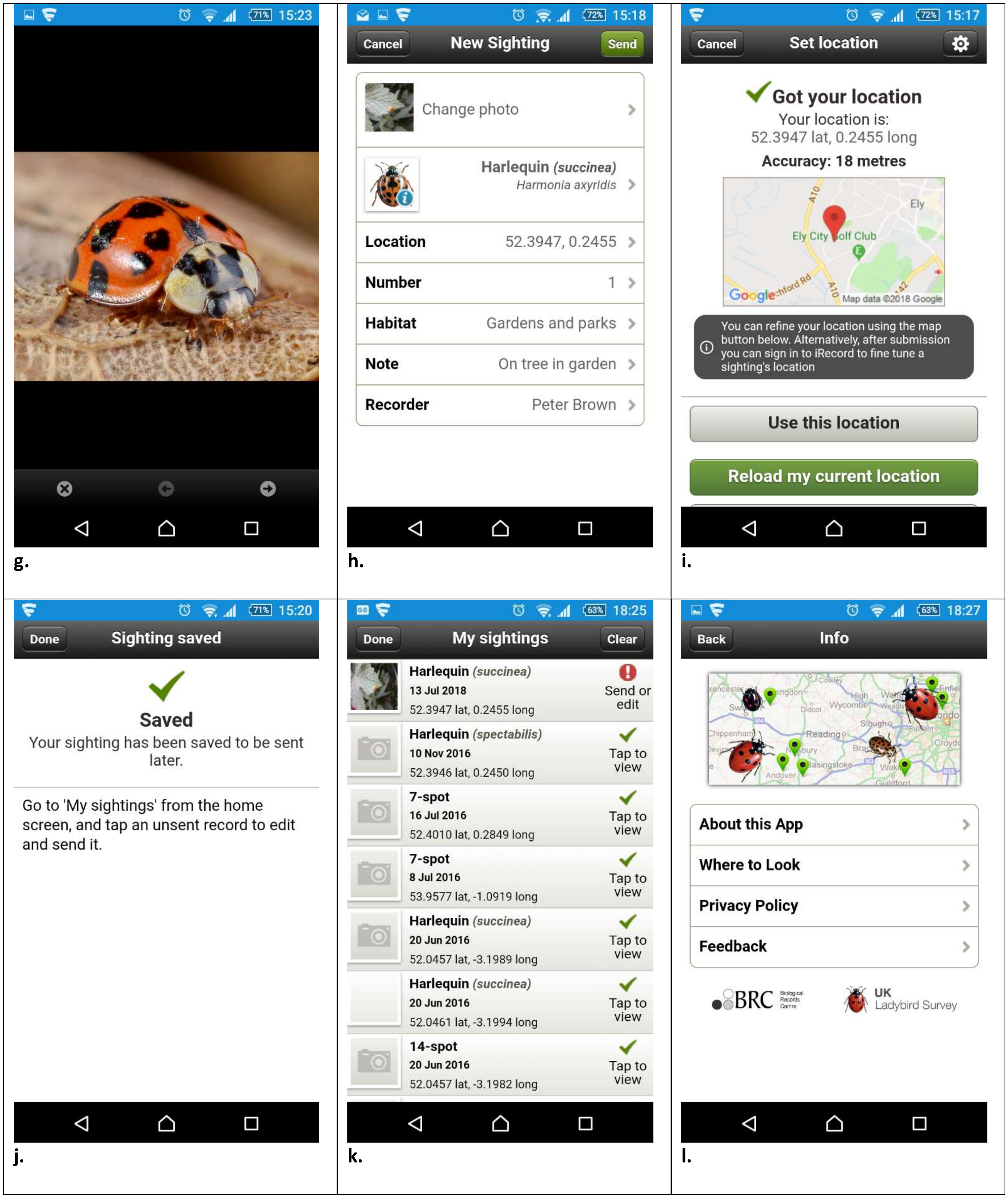

Supplementary Figure 2. Screenshots from the iRecord Ladybirds smartphone application (app) used to record ladybird species, with *Harmonia axyridis* used as an example throughout. Records submitted are checked by the UK Ladybird Survey. **a.** Opening screen; **b.** Ladybird guide: first screen, showing a list of species available to record, ordered by likelihood of occurrence; **c.** Ladybird guide: first screen, showing a list of species available to record, filtered by main colour of the ladybird. In this case the red filter was selected; **d.** Ladybird guide: top of second screen, providing identification information for the species selected; **e.** Ladybird guide: bottom of second screen,

providing further identification and ecological information for the species selected; **f.** Ladybird guide: distribution map for Great Britain of species selected; **g.** Ladybird guide: example gallery image of species selected, enabling the recorder to check their own ladybird against the gallery image(s), prior to record submission; **h.** Adding a record: main recording screen once a species has been selected; **i.** Adding a record: location screen showing map generated from GPS smartphone location; **j.** Adding a record: confirmation screen to show that the record has been saved; **k.** 'My sightings': screen showing records submitted (indicated by green tick) and records saved but not yet submitted (indicated by red circle with exclamation mark); **l.** Information: screen provides background information about the app.
